# Fulminant Toxic Megacolon Without Identifiable Underlying Cause Requiring Emergent Total Colectomy: A Case Report

**DOI:** 10.7759/cureus.104288

**Published:** 2026-02-26

**Authors:** Edgar Alexis Flores García, Hector A Lopez Villicaña, Azael Lopez Lopez, José M Hinojosa Rodríguez, Irvin Hernández Sánchez, Guillermo a Serna Palacios, Melissa Revilla Mora

**Affiliations:** 1 Surgery, Hospital General Nuevo Gómez Palacio, Gómez Palacio, MEX; 2 Surgery, Hospital General de México “Dr. Eduardo Liceaga”, Ciudad de México, MEX; 3 Surgery, Hospital General Universitario “Dr. Joaquín del Valle Sánchez”, Torreón, MEX

**Keywords:** acute colitis, colonic dilatation, exploratory laporotomy, total colectomy, toxic megacolon

## Abstract

Toxic megacolon is a life-threatening condition characterized by acute colonic dilation and systemic toxicity, most commonly associated with inflammatory or infectious colitis. However, ischemic colitis represents an important and underrecognized etiology in elderly patients with significant vascular comorbidities. We report the case of a 71-year-old male with a history of long-standing hypertension, type 2 diabetes mellitus, and chronic kidney disease, receiving losartan, nifedipine, and insulin therapy, who presented with five days of progressive diffuse abdominal pain, severe distension, obstipation, and systemic deterioration. On admission, he was febrile, tachycardic, metabolically acidotic, and demonstrated leukocytosis, elevated inflammatory markers, hyperlactatemia, and renal dysfunction. Initial management included aggressive intravenous fluid resuscitation, broad-spectrum antibiotics, electrolyte correction, and bowel rest. Computed tomography without intravenous contrast revealed severe diffuse colonic dilation with a maximal cecal diameter of 14 cm, without mechanical obstruction or perforation. Given the extreme dilation, systemic toxicity, and early organ dysfunction, urgent surgical intervention was performed within hours of admission. Exploratory laparotomy demonstrated diffuse colonic dilation with mural thinning and serosal inflammatory changes, and total colectomy with end ileostomy was undertaken. Histopathological examination confirmed extensive ischemic colitis with mucosal and submucosal necrosis, without evidence of inflammatory bowel disease or pseudomembranous colitis. The patient required short-term vasopressor support postoperatively but recovered without major complications and was discharged on postoperative day 12. This case emphasizes that toxic megacolon may occur in elderly patients without prior colonic disease and highlights extreme cecal dilation as a critical indicator for early surgical management to improve survival.

## Introduction

Toxic megacolon is a rare but life-threatening complication characterized by acute, nonobstructive colonic dilation associated with systemic toxicity [1]. It most frequently develops as a severe complication of inflammatory bowel disease (IBD), particularly ulcerative colitis, with an estimated incidence of 1%-5% among hospitalized patients with severe colitis, and may also occur secondary to infectious etiologies such as Clostridioides difficile colitis [2,3]. Although advances in medical therapy have reduced its overall incidence, toxic megacolon continues to carry substantial morbidity and mortality. Reported mortality rates range from 8% to 25%, increasing to as high as 40%-50% in cases complicated by perforation [3]. Elderly patients represent a particularly high-risk group due to decreased physiological reserve, comorbidities, and delayed presentation, factors that significantly worsen outcomes.

The pathophysiology involves severe transmural inflammation leading to neuromuscular dysfunction and loss of colonic tone. Nitric oxide has been identified as a key mediator in this process, promoting smooth muscle relaxation and contributing to colonic paralysis and progressive dilation [4]. The resulting distension may rapidly progress to ischemia, perforation, sepsis, and multiorgan failure if not promptly recognized and treated. Diagnosis is based on clinical and radiologic criteria. The diagnostic criteria proposed by Jalan et al. (1969) remain widely used and include radiographic evidence of colonic dilation (>6 cm) plus systemic toxicity manifested by fever (>38.6 °C), tachycardia (>120 bpm), leukocytosis (>10,500 cells/mm³), and anemia, along with at least one of the following: dehydration, altered mental status, hypotension, or electrolyte imbalance [5]. Computed tomography plays a crucial role in confirming colonic dilation and assessing for complications such as perforation or peritonitis.

Early recognition and aggressive medical management are essential; however, failure to improve or clinical deterioration necessitates urgent surgical intervention. Subtotal or total colectomy with end ileostomy remains the standard surgical approach in advanced or refractory cases [3,6]. We present a case of fulminant toxic megacolon in a 71-year-old patient of indeterminate etiology requiring emergent total colectomy. This case is noteworthy due to the absence of an identifiable underlying cause despite thorough evaluation, the rapid clinical deterioration requiring immediate surgical decision-making, and the high-risk profile associated with advanced age. By highlighting diagnostic uncertainty and the need for timely surgical intervention, this report aims to reinforce the importance of early recognition and decisive management in this potentially fatal condition.

## Case presentation

A 71-year-old male with a medical history significant for hypertension for 22 years, type 2 diabetes mellitus for 25 years, and mild chronic kidney disease diagnosed five years earlier presented to the emergency department with a five-day history of diffuse abdominal pain rated 10/10 on the visual analog scale (VAS) [6]. The pain was associated with progressive abdominal distension, nausea, multiple episodes of vomiting, and complete intolerance to oral intake of both liquids and solids. The patient reported absolute constipation and absence of flatus since symptom onset.

His surgical history was notable for an open cholecystectomy performed 23 years earlier. He denied any history of IBD, infectious colitis, colorectal malignancy, or diverticular disease. His chronic medications included losartan 50 mg twice daily, nifedipine 30 mg once daily, and insulin glargine 65 units daily. He denied the use of corticosteroids, opioids, anticholinergic agents, or immunosuppressive drugs.

On admission, the patient appeared acutely ill, dehydrated, and disoriented. Vital signs revealed a temperature of 38.9 °C, heart rate of 137 beats per minute, respiratory rate of 23 breaths per minute, blood pressure of 105/87 mmHg with clinical signs of hypoperfusion, and oxygen saturation of 92% on room air. Peripheral perfusion was decreased with delayed capillary refill. Cardiopulmonary examination demonstrated tachycardia without murmurs and bilaterally diminished breath sounds without focal findings.

Abdominal examination revealed marked distension, diffuse tenderness to palpation, and absence of bowel sounds, without clear initial signs of peritoneal irritation.

Laboratory evaluation showed leukocytosis (18,739 cells/mm³), anemia (hemoglobin 8.6 g/dL), elevated C-reactive protein (121 mg/L), lactate of 3.1 mmol/L, hyponatremia (129 mEq/L), hypokalemia (2.9 mEq/L), elevated creatinine (2.6 mg/dL) with urea of 45 mg/dL, and mild metabolic acidosis (arterial pH 7.33). These findings were consistent with systemic inflammatory response, metabolic derangements, and early organ dysfunction in the setting of systemic toxicity.

**Figure 1:**
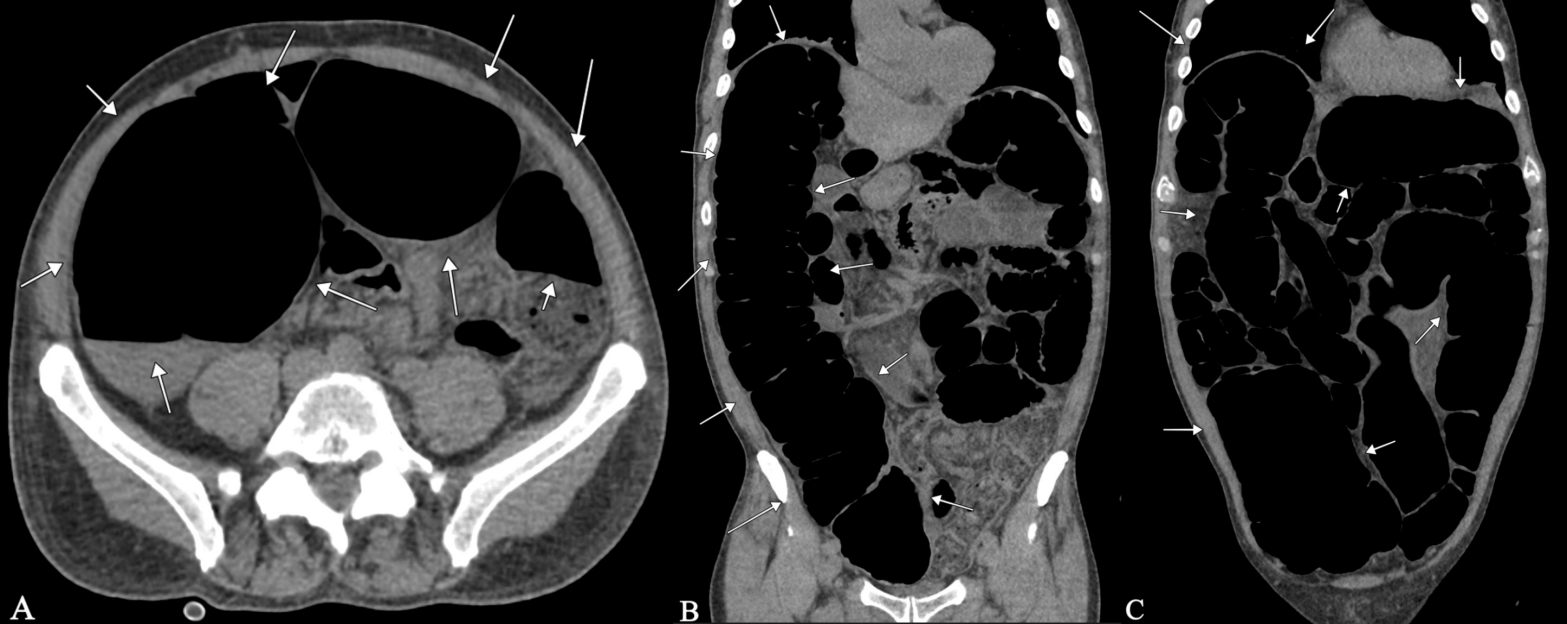
Non-contrast abdominal computed tomography (CT) scan showing severe colonic dilatation. (A) Axial view demonstrating marked dilatation of the ascending colon, with the cecal base indicated by white arrows, measuring approximately 14 cm. (B) Coronal view showing diffuse dilatation of the ascending colon and part of the transverse colon. (C) Axial view at a different level demonstrating extensive colonic dilatation occupying most of the abdominal cavity, with the dilated colon highlighted by white arrows.

Given the severity of the clinical presentation and suspicion of acute abdominal pathology, an abdominal computed tomography (CT) scan was performed (Figure 1). The study was conducted without intravenous contrast due to impaired renal function and underlying chronic kidney disease, in order to avoid additional nephrotoxicity in the emergency setting. CT imaging demonstrated severe and diffuse colonic dilatation, with a maximum cecal diameter of approximately 14 cm. Colonic wall thickening with mild submucosal edema and subtle pericolonic inflammatory changes were observed. No evidence of mechanical obstruction, pneumoperitoneum, pneumatosis intestinalis, intra-abdominal collections, or significant free fluid was identified. The radiologic findings, in correlation with the clinical presentation, were consistent with toxic megacolon.

The preoperative diagnosis was established based on clinical and radiologic integration. The patient met the diagnostic criteria for toxic megacolon as described by Jalan [1].

**Figure 2:**
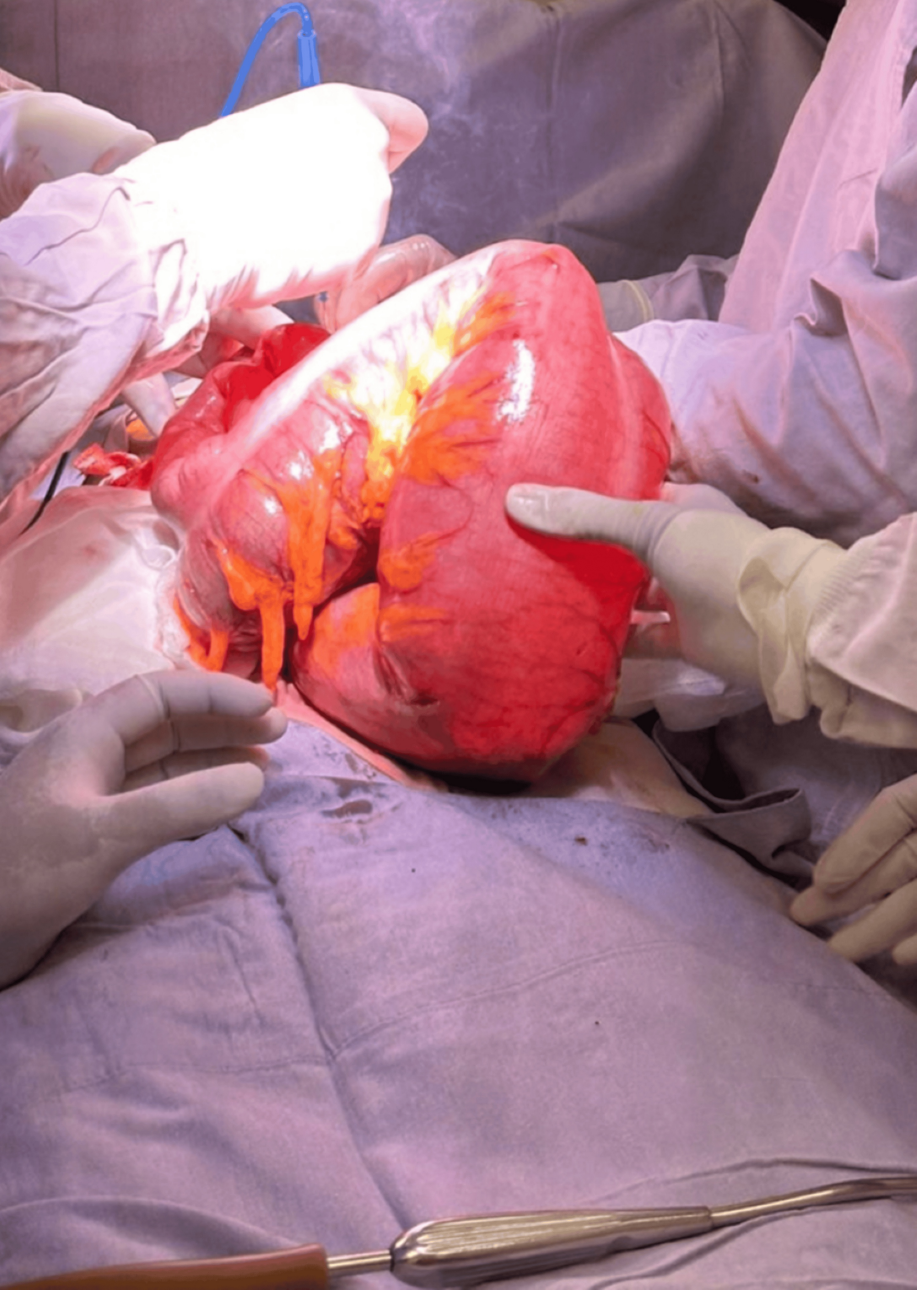
Intraoperative view during exploratory laparotomy showing marked dilatation of the cecal base, measuring approximately 14 cm in diameter.

**Figure 3:**
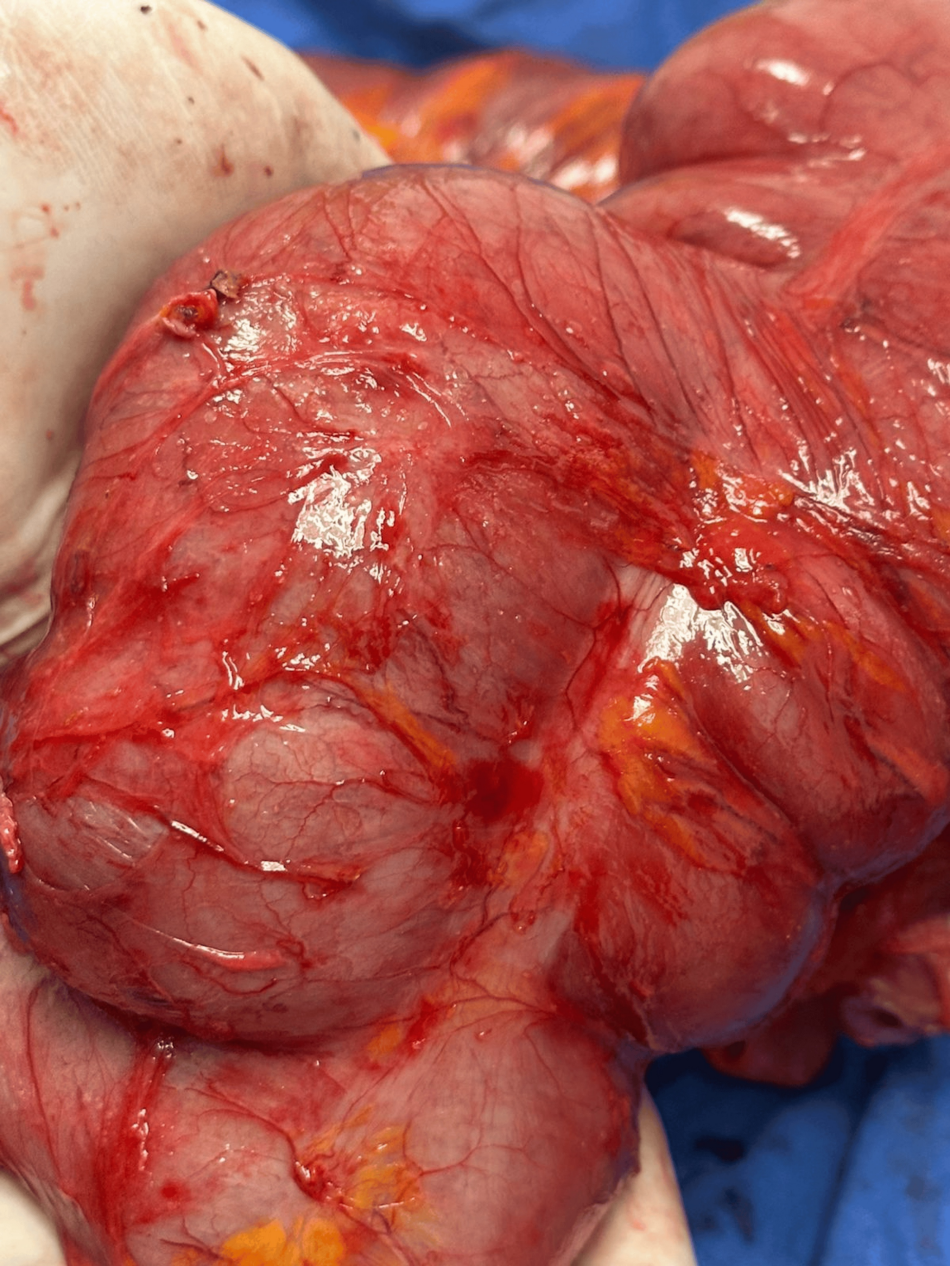
Intraoperative view of the cecum demonstrating marked and pronounced dilatation, consistent with severe colonic distension observed during surgery.

**Figure 4:**
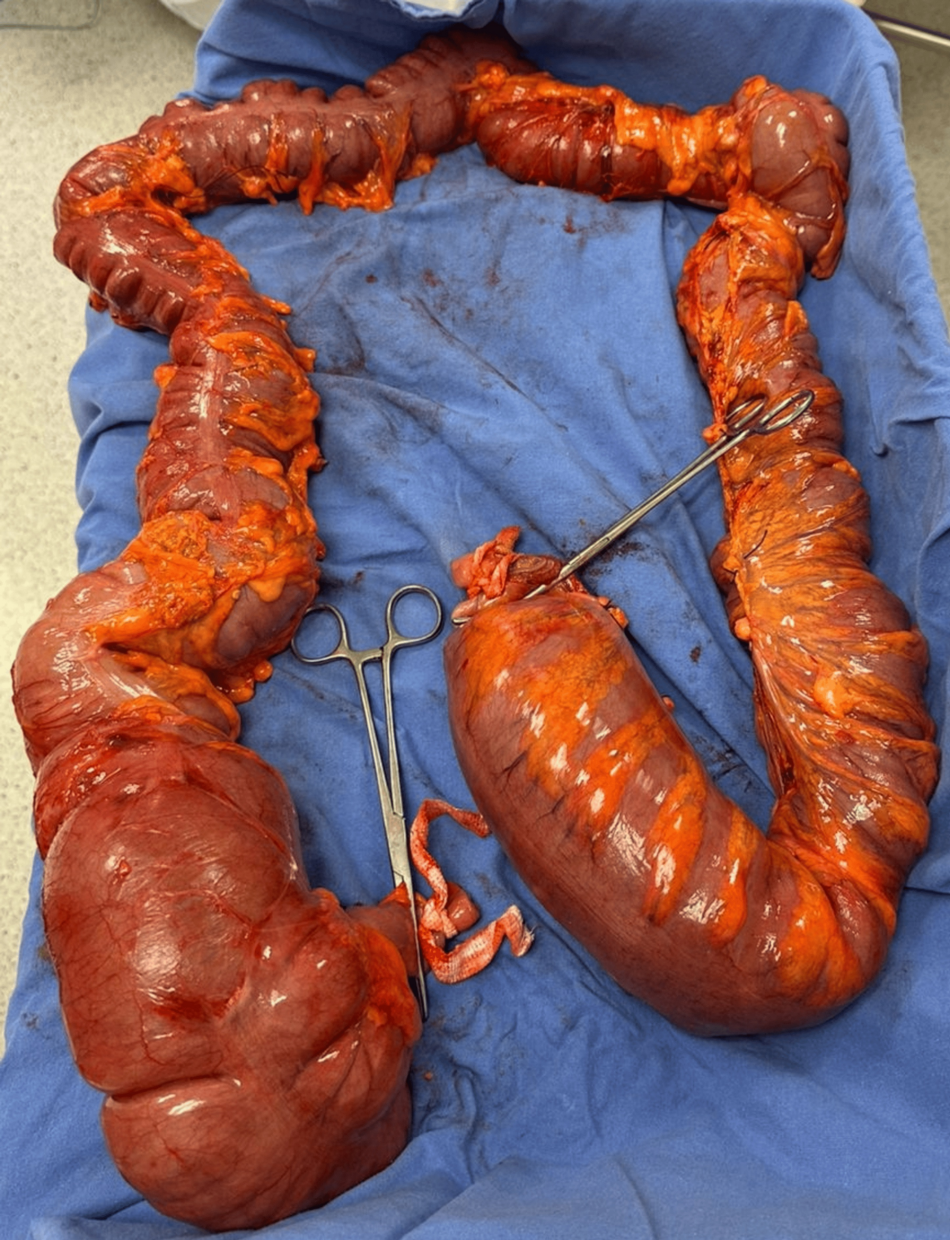
Gross surgical specimen obtained after total colectomy, demonstrating the entire colon from the ileocecal valve through the ascending, transverse, and descending colon, with diffuse and marked dilatation.

Due to progressive clinical deterioration and the imminent risk of perforation, urgent surgical intervention was undertaken. Exploratory laparotomy revealed diffuse colonic dilatation involving the entire colon, including proximal rectal involvement, with marked parietal distension and absence of peristaltic activity. The colonic wall appeared thinned in multiple segments, with areas of vascular congestion and generalized serosal inflammatory changes, indicating diffuse colonic compromise. Given the extensive involvement and the risk of leaving potentially diseased distal bowel, a total colectomy was performed rather than a subtotal colectomy to avoid retaining compromised distal segments. The cecal diameter measured approximately 14 cm (Figures 2-3). A total colectomy with end ileostomy was completed (Figure 4). Total operative time was 165 minutes, with an estimated intraoperative blood loss of 350 mL. Peritoneal fluid and colonic tissue samples were obtained for microbiological analysis, and the entire surgical specimen was sent for histopathological examination.

The patient was transferred to the intensive care unit for hemodynamic and metabolic management. Vasopressor support was required during the first 24 hours, followed by progressive stabilization. Blood cultures and peritoneal fluid cultures were negative. Stool cultures and testing for Clostridioides difficile were also negative.

Histopathological examination revealed extensive ischemic colitis with mucosal and submucosal necrosis, an acute inflammatory infiltrate, and findings consistent with toxic megacolon secondary to colonic ischemia. There was no evidence of inflammatory bowel disease or pseudomembranous colitis.

The postoperative course was favorable, with progressive improvement in inflammatory markers and recovery of renal function. The ileostomy became functional on postoperative day three. No complications such as surgical site infection, anastomotic dehiscence, fistula formation, or prolonged ileus were documented. The patient was transferred to the general surgical ward on postoperative day four and gradually resumed oral intake.

He was discharged on postoperative day 12 in hemodynamically stable condition, tolerating diet, with appropriate ileostomy function, and with outpatient follow-up arranged with general surgery and stoma care services. At the six-week follow-up visit, he demonstrated satisfactory clinical recovery without ostomy-related complications, and delayed intestinal reconstruction was discussed based on clinical evolution and metabolic control.

Written informed consent was obtained from the patient for publication of this case report and accompanying clinical images.

## Discussion

Toxic megacolon represents a severe and potentially fatal complication of colonic inflammation that requires a high index of suspicion and prompt intervention [6]. Although it is classically associated with IBD, particularly ulcerative colitis, it may also occur in the context of infectious colitis, ischemia, or other inflammatory processes [6,7]. In elderly patients, ischemic colitis constitutes a clinically significant and often underrecognized etiology, especially in the presence of substantial vascular comorbidities [7].

In the present case, histopathological examination of the surgical specimen was fundamental in establishing the definitive diagnosis. Macroscopically, the colon was markedly dilated with diffuse mural thinning and areas of serosal congestion. Microscopically, extensive mucosal and submucosal necrosis with acute inflammatory infiltrate and vascular congestion were observed, findings consistent with severe ischemic colitis [8]. No chronic inflammatory architectural distortion suggestive of IBD was identified, and no pseudomembranes were observed. These findings confirmed toxic megacolon secondary to extensive colonic ischemia.

A structured differential diagnosis was carefully considered. Infectious colitis, particularly Clostridioides difficile, represents a common cause of toxic megacolon [9]; however, stool testing and microbiological cultures were negative in this patient. IBD was considered unlikely given the absence of prior gastrointestinal symptoms and the lack of chronic histopathological changes. Mechanical obstruction was excluded both radiologically and intraoperatively. The presence of long-standing hypertension, diabetes mellitus, and chronic kidney disease strongly supported an ischemic etiology, likely related to underlying vascular compromise and hypoperfusion [8].

Advanced age plays a critical role in both clinical presentation and outcomes. Patients older than 70 years frequently exhibit a more insidious course and may present at later stages of disease, increasing the risk of complications [10]. Moreover, elderly patients have been shown to experience higher postoperative morbidity and mortality compared with younger populations [9]. Reported overall mortality rates for toxic megacolon range from 19% to 40%, rising to over 50% in cases complicated by perforation or delayed surgical intervention [11]. These data underscore the importance of early recognition and timely operative management, particularly in high-risk elderly patients such as the one described.

Although medical management, including bowel rest, intravenous fluids, broad-spectrum antibiotics, and corticosteroids when indicated, constitutes first-line therapy in hemodynamically stable patients [6], it was not considered appropriate in this case. The patient presented with severe systemic toxicity, significant metabolic derangements, progressive clinical deterioration, and extreme colonic dilation measuring 14 cm at the cecum, well above the 12 cm threshold associated with increased risk of perforation [9]. Additionally, elevated lactate levels and early organ dysfunction indicated ongoing hypoperfusion. In this context, delaying surgery in favor of conservative therapy would likely have increased the risk of perforation and septic shock. Therefore, immediate surgical intervention was deemed the safest and most appropriate course of action.

Surgical management remains the cornerstone of treatment in advanced cases. Subtotal or total colectomy with end ileostomy is widely regarded as the procedure of choice [11], as it effectively removes the diseased colon while minimizing operative time and postoperative complications. Early surgical intervention has consistently been associated with improved survival and reduced rates of perforation and sepsis. In this patient, the decision to perform total colectomy was justified by diffuse colonic involvement, including proximal rectal compromise, thereby avoiding retention of potentially ischemic distal segments.

This case highlights ischemic colitis as a significant cause of toxic megacolon in elderly patients with multiple vascular risk factors. It also emphasizes the importance of integrating clinical, radiologic, microbiologic, and histopathological findings to establish the definitive etiology. Early surgical management in the setting of advanced dilation and systemic toxicity may significantly improve outcomes, even in high-risk geriatric patients.

## Conclusions

This case underscores that extreme colonic dilation, particularly cecal diameters reaching 14 cm, represents a critical radiologic finding that should prompt immediate surgical evaluation due to the high risk of perforation and mortality. Significant colonic dilation in the presence of systemic toxicity warrants urgent decision-making, even in the absence of a previously established colonic diagnosis.

Furthermore, toxic megacolon may develop in elderly patients without a prior history of IBD or infectious colitis, making ischemic colitis an important and potentially underrecognized etiology in this population. The absence of a known underlying condition should not delay intervention when clinical and radiologic findings indicate impending catastrophic progression.

Early recognition of extreme dilation combined with decisive surgical management remains essential to improving survival in high-risk geriatric patients.
